# Acute RhoA/Rho Kinase Inhibition Is Sufficient to Restore Phagocytic Capacity to Retinal Pigment Epithelium Lacking the Engulfment Receptor MerTK

**DOI:** 10.3390/cells10081927

**Published:** 2021-07-29

**Authors:** Yingyu Mao, Silvia C. Finnemann

**Affiliations:** Center for Cancer, Genetic Diseases, and Gene Regulation, Department of Biological Sciences, Larkin Hall, Fordham University, 441 East Fordham Road, Bronx, NY 10458, USA; yydmao@gmail.com

**Keywords:** F-actin, MerTK, phagocytosis, TAM receptors, retinal pigment epithelium, RhoA, ROCK

## Abstract

The diurnal phagocytosis of spent photoreceptor outer segment fragments (POS) by retinal pigment epithelial (RPE) cells is essential for visual function. POS internalization by RPE cells requires the assembly of F-actin phagocytic cups beneath surface-tethered POS and Mer tyrosine kinase (MerTK) signaling. The activation of the Rho family GTPase Rac1 is necessary for phagocytic cup formation, and Rac1 is activated normally in MerTK-deficient RPE. We show here that mutant RPE lacking MerTK and wild-type RPE deprived of MerTK ligand both fail to form phagocytic cups regardless of Rac1 activation. However, in wild-type RPE in vivo, a decrease in RhoA activity coincides with the daily phagocytosis burst, while RhoA activity in MerTK-deficient RPE is constant. Elevating RhoA activity blocks phagocytic cup formation and phagocytosis by wild-type RPE. Conversely, inhibiting RhoA effector Rho kinases (ROCKs) rescues both F-actin assembly and POS internalization of primary RPE if MerTK or its ligand are lacking. Most strikingly, acute ROCK inhibition is sufficient to induce the formation and acidification of endogenous POS phagosomes by MerTK-deficient RPE ex vivo. Altogether, RhoA pathway inactivation is a necessary and sufficient downstream effect of MerTK phagocytic signaling such that the acute manipulation of cytosolic ROCK activity suffices to restore phagocytic capacity to MerTK-deficient RPE.

## 1. Introduction

In the mammalian retina, photoreceptor neurons continuously renew their light-sensitive outer segments to maintain retinal function for life. The addition of new membrane disks at the proximal end of outer segments is precisely balanced with the diurnal shedding of distal spent outer segment tips followed by immediate phagocytosis by the adjacent retinal pigment epithelium (RPE) [1,2]. Life-long circadian outer segment renewal is thought to prevent cumulative damage acquired with age by photoreceptor and RPE cells, as both cell types are post-mitotic and permanent in the mammalian eye. On the other hand, the incomplete phagolysosomal digestion of outer segment debris causes the formation of oxidized indigestible proteins and lipids as lipofuscin in RPE cells, and much evidence suggests that lipofuscin components are detrimental to RPE cell viability and function [3]. Lipofuscin components enhance photo-oxidative stress, which in turn harms RPE cells in many ways, including by triggering cell death, damaging mitochondrial and nuclear DNA, and altering gene expression [4,5]

The molecular mechanism of outer segment renewal is only partly understood. The mechanism of POS phagocytosis by RPE cells is a specialized form of efferocytosis [6]. Its regulation likely involves RPE ion channel activity, although specific contributions remain to be established [7,8,9,10]. Shedding photoreceptor outer segment fragments (POS) are marked by the exposure of phosphatidylserine, which serves to signal the need for POS engulfment to adjacent RPE cells [11]. Extracellular phosphatidylserine-binding proteins MFG-E8, Gas6, and Protein S in the subretinal space between RPE cells and photoreceptor outer segments serve to activate at least two phagocytic receptors in the RPE cells: apical αvβ5 integrin receptors are stimulated by MFG-E8 to signal towards the F-actin regulator GTPase Rac1 and, separately, to the receptor tyrosine kinase Mer (MerTK) via focal adhesion kinase [12,13,14,15]. Full MerTK activation also requires the engagement of its extracellular ligands, with Protein S and Gas6 physiologically relevant to MerTK function in RPE phagocytosis in vivo [16].

MerTK is a member of the Tyro3/Axl (also known as UFO)/MerTK (TAM) family of highly related receptor tyrosine kinases. Mutations in MerTK can cause autosomal recessive forms of retinitis pigmentosa with numerous mutations associated with the disease spanning the entire *mertk* gene [17,18,19]. Studies on mutant MerTK expression or function in retinitis pigmentosa patients have yet to be reported. In the Royal College of Surgeons (RCS) rat strain, a naturally occurring mutation in the gene encoding MerTK abolishes *mertk* gene function, and no MerTK protein can be detected [20,21]. RCS RPE cells fail to engulf POS, leading to the accumulation of outer segment debris in the subretinal space, photoreceptor death and retinal degeneration [22]. Mice in a mixed genetic background in which the *mertk* gene has been disrupted by the insertion of a neomycin expression cassette such that no MerTK protein can be detected show the same dramatic retinal degeneration as the RCS rat [23]. However, the same *mertk* gene disruption does not cause retinal degeneration in mice of a pure C57BL6 genetic background with high RPE expression of Tyro3, suggesting that Tyro3 can substitute for MerTK in RPE function [23,24]. Downstream signaling stimulated by MerTK ligation is complex and may include Src family kinase and phospholipase involvement [25,26]. However, it remains poorly understood which specific aspects of the MerTK signaling response in the RPE are required for POS engulfment.

POS engulfment is F-actin dependent, such that it requires the de novo assembly of an F-actin structure beneath surface-bound POS that is commonly referred to as the “phagocytic cup” [27]. We previously showed that activation of the Rho family GTPase Rac1 is required to assemble a functional phagocytic cup for POS internalization [14]. Rac1 activation is impaired in RPE cells lacking αvβ5 integrin but occurs to a normal extent in response to POS recognition/binding in RPE cells lacking engulfment receptor MerTK [14]. Here, we show that RPE cells cannot form phagocytic cups beneath surface-tethered POS in the absence of MerTK signaling, which implies roles for F-actin-regulating mechanisms in phagocytosis other than and in addition to Rac1 activation. Moreover, we find that POS engulfment by wild-type phagocytic RPE cells in vivo is associated with a decrease in the GTP load of the small GTPase RhoA, which is indicative of RhoA inhibition. In contrast, RhoA activity does not fluctuate in phagocytosis-defective RPE in vivo. Using well-characterized pharmacological agents to manipulate Rho GTPases or their direct downstream mediators, Rho kinases (ROCKs), we find that activating and inhibiting the RhoA pathway acutely during the phagocytosis process inhibits or promotes F-actin recruitment and POS internalization, respectively. Altering the cytosolic RhoA signaling pathway is sufficient to promote or inhibit internalization and subsequent phagosome acidification irrespective of the presence of MerTK ligands or MerTK receptors themselves. Remarkably, acute ROCK inhibition is sufficient to trigger the engulfment of endogenous POS by MerTK-deficient RPE, suggesting that RhoA/ROCK pathway inhibition is both sufficient and necessary to overcome MerTK deficiency.

## 2. Materials and Methods

Materials were purchased from Sigma-Millipore (St. Louis, MO, USA) or Thermofisher (Waltham, MA, USA), unless otherwise stated.

### 2.1. Animals

Animals were handled according to the ARVO Guide for the Use of Animals in Vision Research and the Guide for the Care and Use of Laboratory Animals (NIH, 8th edition) and reviewed and approved by the Fordham University Institutional Animal Care and Use Committee (protocol B08-01R). Pink-eyed dystrophic RCS (rdy/rdy-p) rats and wild-type (wt) Sprague Dawley rats were housed in 12 h dark:12 h light cycles and were fed standard rodent diet 5053 and drinking water ad libitum. Animals of both sexes were used. For tissue harvest, rats were sacrificed by CO_2_ asphyxiation followed by cervical dislocation. Eyeballs were enucleated and processed immediately post mortem. To prepare primary RPE, 9–11-day-old rats were sacrificed between 1 and 3 h after light onset followed by processing as described in Section 2.3. To prepare the eyecups for immunoblotting, 21–24-day-old rats were sacrificed at specific times before or after light onset as required by the experiment. For the isolation of eyecups for whole mount preparation, 15–18-day-old rats were sacrificed 5 min after light onset. Cornea, lens, and retina were dissected from the eyes before the immediate lysis of posterior eyecups and analysis as in Section 2.7, or further radial cuts to flatten eyecups followed by incubation for 2 h with or without pharmacological reagents in a humidified atmosphere at 37 °C and 5% CO_2_. Eyecups were then either live-stained with 0.4 μM LysoTracker^®^ Green DND-26 (#L7526, Thermofisher) in FluoroBrite™ DMEM (A1896701, Thermofisher) for 15 min and imaged live as described previously [28], or fixed in 4% paraformaldehyde (#15710, Electron Microscopy Sciences) in PBS and immunofluorescence labeling with rhodopsin antibody B6-30 [29] (#NBP2-25160, Novus Biologicals, 0.1 μg/mL in PBS) and AlexaFluor-conjugated secondary antibodies (#A21202, Thermofisher, 1:300 in PBS) followed by nuclei counterstain for 15 min at room temperature with 4′,6-diamidino-2-phenylindole (DAPI, #62248, Thermofisher, 0.5 μg/mL in PBS) [30]. Images of F-actin and POS were acquired on a Leica TSP5 laser scanning confocal microscopy system using the sequential scanning mode and compiled using Adobe Photoshop CS4. DAPI nuclei counterstain was used to aid in sample observation but was not routinely imaged to avoid the excessive bleaching of stainings.

### 2.2. GTPase Activity Assays

RhoA GLISA GTPase activity assays (#BK124, Cytoskeleton Inc., Denver, CO, USA) were carried out following the manufacturer’s protocol on freshly lysed posterior eyecups collected at different times of day. Two eyecups from the same animal were pooled for each sample.

### 2.3. Primary RPE Cell Culture

RPE cells from 9–11-day-old wt or RCS rats for primary culture were isolated as previously published in detail [12]. In brief, the cornea, lens, iris, and vitreous body were removed from freshly enucleated eyes. Eyecups were incubated in 1 mg/mL hyaluronidase (#H3506, Sigma-Millipore) in Hank’s balanced saline solution without Ca^2+^ and Mg^2+^ for 45 min at 37 °C. The neural retina was removed, and eyecups were further incubated in 2 mg/mL trypsin (#27250018, Thermofisher) in Hank’s balanced saline solution with Ca^2+^ and Mg^2+^ for 30 to 45 min at 37 °C. RPE sheets were manually collected from the underlying choroid. Purified RPE patches were grown in 96-well plates with collagen IV (#354233, Corning)-coated glass coverslips in DMEM (#D6429, Sigma-Millipore) 10% FBS (#F4135, Sigma-Millipore) at 37 °C, 5% CO_2_ for 5–7 days before experiments.

### 2.4. POS Phagocytosis Assays

POS were purified from freshly obtained porcine eyes from a local slaughterhouse according to established procedures [31]. Fluorescent dye labeling was performed immediately before use as described with 0.1 mg/mL FITC (FITC Isomer I, #F1906, Thermofisher), or 0.01 mg/mL Texas Red-X (mixed isomers, #T6134, Thermofisher) [31]. Synchronized phagocytosis assays were performed as described in detail previously [32]. In brief, cells on glass coverslips in multi-well plates were fed POS at a density of 10 particles/cell in DMEM supplemented with 1.25 μg/mL recombinant mouse MFG-E8 (#2805-MF-50/CF, R & D Systems, Minneapolis, MN, USA) for the duration of experiments at 37 °C, or for 1 h at 20 °C to allow only POS binding in synchronized phagocytosis assays [33]. Cells with surface-bound POS were washed twice with serum-free DMEM after the 20 °C binding phase before further incubation with DMEM with or without 2 μg/mL protein S (#APP012A, Aniara, West Chester, OH, USA) at 37 °C. All phagocytosis assays were terminated with three washes of phosphate buffered saline (PBS) supplemented with 0.1 mM CaCl_2_ and 1 mM MgCl_2_ followed by live imaging or fixation with 4% PFA in PBS.

### 2.5. RhoA Pathway Manipulations in RPE in Culture and RPE Ex Vivo

To activate RhoA, primary RPE cells were incubated with cell-permeable exoenzyme RhoA activator that has been shown to activate Rho specifically [34]. RhoA activator (#CN03, Cytoskeleton Inc) was applied to RPE cells in culture at 1 μg/mL in DMEM 10% FBS for 3 h directly prior to POS phagocytosis assays. The widely used and well-characterized ROCK-specific competitive inhibitor Y-27632 (#1254, Tocris Bioscience, Minneapolis, MN, USA, [35]), was applied at 25 μM during POS challenge for cell culture assays. Initial experiments established similar effects on promoting POS internalization by wt RPE in the absence of MerTK ligand for Y-27632 at 50 μM, and efficacy of other ROCK inhibitors, H-1152 (#2414, Tocris Bioscience) and HA-1100 (#2415, Tocris Bioscience), used at 5 μM and 50 μM, respectively (Supplementary Figure S1).

To induce phagocytosis by RPE tissue ex vivo, posterior RCS rat eyecups dissected 5 min after light onset were incubated in DMEM with 10% FBS and with and without 50 μM Y-27632 for 2 h followed by labeling and immediate live imaging as described in Section 2.1.

### 2.6. Immunofluorescence, F-Actin and Live Lysosome Microscopy

For immunofluorescence and F-actin staining, RPE cells were fixed with 4% paraformaldehyde in PBS for 20 min. All further incubations used PBS supplemented with 0.1 mM CaCl_2_ and 1 mM MgCl_2_ as solvent. For the selective staining of surface-bound POS, cells were incubated without permeabilization with anti-rhodopsin antibody B6-30 and secondary AlexaFluor488-conjugated antibody as described in Section 2.1. For Texas Red-labeled POS and F-actin co-staining, fixed cells were permeabilized with 0.5% Triton-X100 for 15 min before incubation with AlexaFluor488-conjugated phalloidin (#A12379, Thermofisher, 1:100). Nuclei were co-stained with DAPI as described in Section 2.1. For Texas Red-labeled POS and acidified organelle co-staining, cells were live labeled with 0.4 μM LysoTracker^®^ Green DND-26 in FluoroBrite™ DMEM at 37 °C for 15 min followed by immediate live imaging. Images were acquired on a Leica TSP5 laser scanning confocal microcopy system using the sequential scanning mode and compiled using Adobe Photoshop CS4.

Images were processed using Image J to quantify signals from bound POS (AlexaFluor488-positive, Texas Red positive), total POS (Texas Red positive), internal POS (Texas Red positive, AlexaFluor488 negative), or acidified, internal POS (TexasRed positive, LysoTracker^®^ positive), or to quantify the number of POS associated with F-actin.

### 2.7. SDS-PAGE and Immunoblotting

Individual dissected posterior eyecups were lysed in 160 μL of HNTG detergent buffer (50 mM HEPES, pH 7.4, 150 mM NaCl, 10% glycerol, 1.5 mM MgCl_2_, 1% Triton X-100) freshly supplemented with 1% protease and phosphatase inhibitor cocktails (#P8340, Sigma-Millipore, and #78420, Thermofisher, respectively). Cleared lysates representing equal eyecup tissue fractions were separated on 12% SDS polyacrylamide gels using a standard Tris–glycine buffer system (Novex gels and solutions, Thermofisher). Proteins were transferred to nitrocellulose membranes (#88018, Thermofisher) for immunodetection with primary and appropriate horseradish peroxidase (HRP)-conjugated secondary antibodies. The primary antibodies used were porin (#4866, Cell Signaling, 1:5,000), RhoA (#sc-179, Santa Cruz Biotechnologies, Santa Cruz, CA, USA, 1:200), rhodopsin (clone B6-30, 0.01 μg/mL), and α-tubulin (#9099, Cell Signaling, 1:5,000). The secondary antibodies used were donkey-anti-rabbit IgG-HRP (#16029, Thermofisher, 1:10,000) and donkey-anti-mouse IgG-HRP (#16011, Thermofisher, 1:5,000). Chemiluminescence reaction signals from ECL-plus substrate (#NEL105001EA, Perkin-Elmer, Shelton, CT, USA) were captured with X-ray films (#3018, Denville Scientific, Swedesboro, NJ, USA), which were scanned and quantified by densitometry using Image Quant^TM^ TL 7.0 (GE Healthcare, Chicago, IL, USA).

### 2.8. Statistical Analysis

All experiments were performed at least three times independently. All values are presented as the mean ± SD. Statistical analyses were performed using Prism 7 (Graphpad, San Diego, CA, USA). Differences with *p* < 0.05 were considered statistically significant. The specifics of the statistical analyses for each experiment are provided in the figure legends.

## 3. Results

### 3.1. Recruitment of F-Actin Phagocytic Cups Beneath Surface-Bound POS Requires MerTK Receptor Ligation

**Figure 1:**
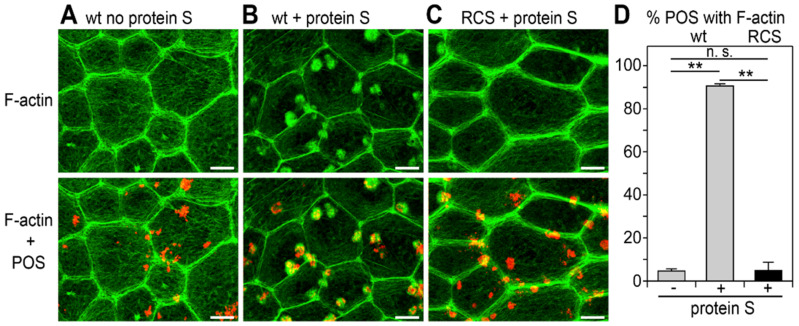
Recruitment of F-actin phagocytic cups beneath bound POS by RPE cells requires MerTK ligation. Primary wt (**A**,**B**) or RCS RPE cells (**C**) were challenged with Texas Red-labeled POS in a synchronized phagocytosis assay. Cells with pre-bound POS were incubated at 37 °C for 10 min with serum-free DMEM (**A**) or DMEM supplemented with MerTK ligand Protein S (**B**,**C**) before fixation and F-actin labeling. Fields show maximal projections of F-actin only (upper row) and overlay of F-actin (green) and POS (red) (lower row). Scale bars, 5 μm. Fields shown are representative areas of larger fields that were imaged and quantified. Please see Supplementary Figure S2 for example fields with nuclei counterstain illustrating that cortical F-actin is indicative of cell distribution. (**D**) Bars show fraction of F-actin-associated POS of total POS obtained from samples as shown in (**A**–**C**) expressed as mean ± SD; *n* = 3 independent experiments with 4 random fields of ~25 cells counted in each experiment. Gray bars, wt cells; black bar, RCS cells. ** indicates *p* < 0.01 according to the Kruskal–Wallis test and Dunn’s post hoc comparison; n.s., not significant, *p* > 0.05.

Signaling by the small GTPase Rac1 is necessary for F-actin recruitment beneath surface-bound POS and for POS engulfment [14]. RPE cells in the mutant RCS rats lacking functional MerTK still activate Rac1 during POS binding like wt RPE cells, but fail to engulf surface-tethered POS. To assess if MerTK activity is required for F-actin assembly beneath bound POS, we used a synchronized POS phagocytosis assay testing unpassaged, differentiated primary rat RPE cells in culture. Adding purified MerTK ligand protein S was necessary to promote the recruitment of F-actin phagocytic cups beneath surface-bound POS in wt RPE cells (Figure 1A,B,D). We noted that lateral F-actin appeared somewhat more condensed in wt RPE cells in response to POS with protein S, a phenomenon that will require further study. MerTK-deficient RCS RPE cells failed to assemble F-actin beneath bound POS despite the presence of protein S (Figure 1C,D). Thus, MerTK receptor engagement subsequent to POS binding is necessary for the recruitment of F-actin beneath bound POS and for POS engulfment.

### 3.2. MerTK-Deficient RPE In Vivo Lacks a Dip in RhoA Activity that Coincides with the Peak of Diurnal POS Phagocytosis in wt RPE

**Figure 2:**
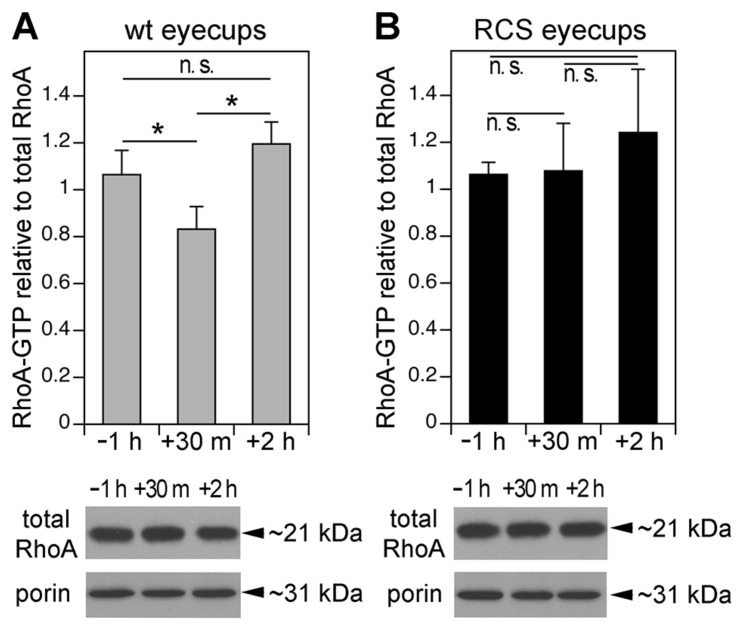
Activity of the GTPase RhoA decreases after light onset in vivo in wt but not MerTK-deficient RCS RPE tissue. Wt (**A**) and RCS (**B**) posterior eyecups enriched in RPE were collected 1 h before, 30 min, and 2 h after light onset and analyzed by RhoA G-LISA and immunoblotting. RhoA-GTP values were normalized to RhoA protein in each sample. Representative RhoA immunoblots beneath bar graphs show that RhoA did not vary with time of day regardless of genotype. Porin re-probing was used to confirm sample protein load. Full blot membrane images are shown in Supplementary Figure S3. Bars show active RhoA-GTP relative to total RhoA amount in each sample expressed as mean ± SD; *n* = 4 independent experiments; *, *p* < 0.05 according to the Kruskal–Wallis test and Dunn’s post hoc comparison; n.s., not significant, *p* > 0.05.

Phagocytic cups consist of dynamic F-actin structures commonly promoted by Rac1 or Cdc42 signaling. These felt-like F-actin structures differ from the robust, parallel F-actin fibers present in stress fibers that are promoted by RhoA signaling. We therefore suspected that RhoA activity might be detrimental to phagocytic cup formation. We thus measured RhoA-GTP load, which is directly indicative of RhoA activity, at the time of peak RPE phagocytosis in the eye. RhoA-GTP in wt RPE cells in vivo decreased briefly after light onset, with levels 28% lower than levels 1 h before light onset, and 43% lower than levels 2 h after light onset, just after the diurnal peak of POS phagocytosis (Figure 2A). In contrast, RhoA-GTP levels did not vary among these times of day in RCS RPE cells in vivo in which no phagocytosis occurs (Figure 2B). The RhoA GTP load changes reflected changes in its GTPase activity regulation rather than protein expression regulation, as the total RhoA expression remained the same at all time points tested in both wt and MerTK-deficient RPE cells (Figure 2A,B, Western blot panels). These results show that decreased RhoA activity directly correlates with POS phagocytosis in vivo.

### 3.3. ROCK Inhibition Rescues Phagocytic Cup Formation by RPE Cells in Which RhoA Is Inhibited

**Figure 3:**
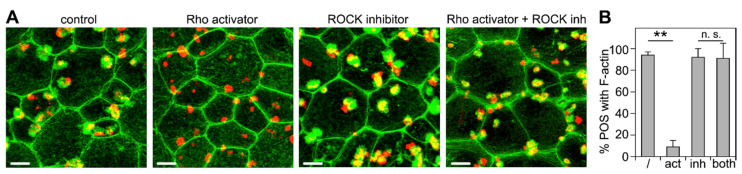
ROCK inhibition rescues inhibition of F-actin phagocytic cup formation by RhoA activator. (**A**) Primary wt RPE cells were challenged with Texas Red-labeled POS in a synchronized phagocytosis assay. Cells with pre-bound POS were incubated at 37 °C for 10 min with DMEM supplemented with MerTK ligand Protein S plus DMSO solvent (control), RhoA activator, ROCK inhibitor, or both before fixation and F-actin labeling. Fields show maximal projections of overlays of F-actin (green) and POS (red). Fields shown are representative areas of larger fields that were imaged and quantified. Scale bars, 5 μm. (**B**) Bars show fraction of F-actin-associated POS of total POS obtained from samples as shown in (**A**) expressed as mean ± SD; *n* = 3 independent experiments with 4 random fields of ~25 cells counted in each experiment. ** indicates *p* < 0.01 according to the Kruskal–Wallis test and Dunn’s post hoc comparison; n.s., not significant, *p* > 0.05.

Next, we set out to directly test the effects of manipulating RhoA on F-actin phagocytic cup formation. The activation of RhoA was achieved by pre-treating wt primary RPE cells in culture with an engineered cell-permeable bacterial cytotoxic necrotizing factor (CNF). The CNF toxin activates Rho GTPase isoforms (RhoA, RhoB, and RhoC) by delaminating glutamine-63, which renders a constitutively active Rho GTPase [34]. Its specificity for Rho GTPase activation is superior to other compounds known to activate Rho GTPases, which also directly target other pathways, e.g., lysophosphatidic acid and calpeptin, both of which are involved in the activation and inhibition of other pathways [36,37]. Strikingly, unlike wt control cells, Rho activator-treated wt RPE cells failed to form phagocytic cups beneath bound POS (Figure 3A,B). Inhibiting ROCKs using the well-characterized inhibitory compound Y-27632 had no effect on F-actin phagocytic cup formation (Figure 3A,B). However, ROCK inhibition was fully effective in preventing the inhibitory effect of Rho activator pre-treatment (Figure 3A,B). Notably, Rho activator pretreatment was performed prior to the addition of POS, while ROCK inhibitor was added only at the time of POS challenge. As expected for unpassaged primary RPE cell cultures, cell appearance showed some degree of variability across samples and experiments. Yet, we did not note consistent gross changes in cell size or shape in response to any of the treatments. Altogether, our results suggest that active RhoA inhibits phagocytic cup formation through ROCK.

### 3.4. Inhibition of the RhoA-ROCK Pathway Restores Phagocytic Cup Formation and POS Internalization into Acidified Phagolysosomes to RPE Cells that Lack MerTK Activity

**Figure 4:**
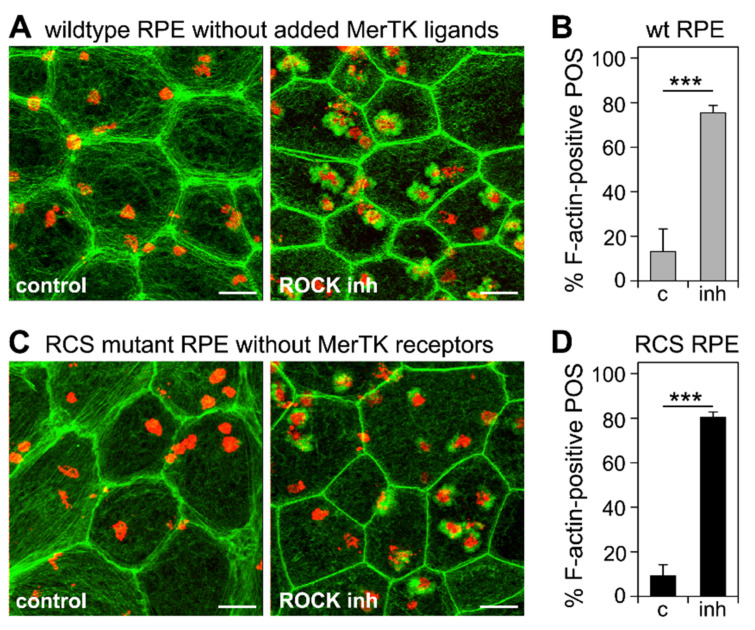
ROCK inhibition is sufficient to induce F-actin phagocytic cup assembly in wt RPE cells deprived of MerTK ligand protein S and in RCS RPE cells lacking MerTK receptors. Primary RPE cells were challenged with Texas Red-labeled POS in a synchronized phagocytosis assay. Cells with pre-bound POS were incubated at 37 °C for 10 min with DMEM with DMSO solvent (control), or ROCK inhibitor as indicated before fixation and F-actin labeling. Fields in (**A**,**C**) show maximal projections of overlays of F-actin (green) and POS (red). Fields shown are representative areas of larger fields that were imaged and quantified. Scale bars, 5 μm. (**A**) Fields show wt RPE cells. (**B**) Bars show fraction of F-actin-associated POS of total POS in wt RPE cells deprived of MerTK ligand obtained from samples as shown in (**A**). (**C**) Fields show RCS mutant RPE cells. (**D**) Bars show fraction of F-actin-associated POS of total POS in MerTK receptor-deficient RPE obtained from samples as shown in (**C**). Data are expressed as mean ± SD; *n* = 3 independent experiments with 4 random fields of at least 25 cells each counted in each experiment. *** indicates *p* < 0.001 according to Student’s *t*-test for pairwise comparison of averages from each experiment (*n* = 3).

Next, we tested whether inhibiting the RhoA-ROCK signaling pathway may substitute for MerTK signaling and induce POS internalization. To this end, we performed synchronized POS phagocytosis assays on wt RPE cells deprived of MerTK ligand and on RCS RPE cells lacking MerTK receptors. Notably, ROCK inhibitor did not alter POS binding by either wt or MerTK-deficient RCS RPE cells (Supplementary Figures S4 and S5). However, the labeling of F-actin 30 min after the incubation of cells with pre-bound POS at a permissive temperature revealed that ROCK inhibitor was sufficient to promote F-actin phagocytic cup assembly beneath surface-tethered POS by either cell type lacking MerTK signaling (Figure 4A,C). Quantification revealed that the fraction of bound POS associated with F-actin cups increased from 13% to 75% on average in wt RPE cells lacking MerTK ligand (Figure 4B) and from 9% to 80% in RCS RPE cells lacking MerTK receptors (Figure 4D).

**Figure 5:**
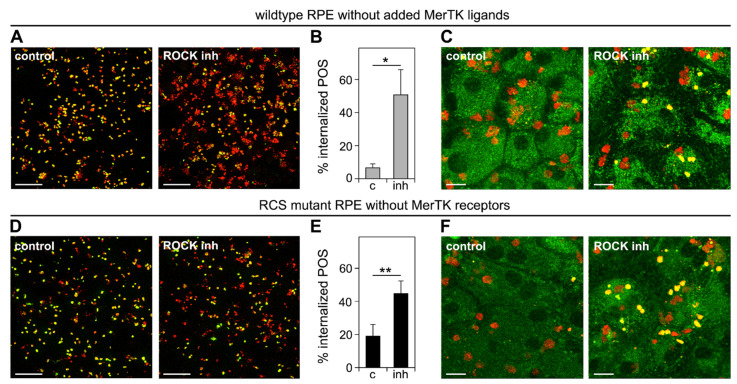
ROCK inhibition is sufficient to promote engulfment of POS into acidified phagolysosomes by wt RPE cells deprived of MerTK ligand protein S and by RCS RPE cells lacking MerTK receptors. Primary wt (**A**–**C**) and RCS (**D**–**F**) RPE cells were challenged with Texas Red-labeled POS in a synchronized phagocytosis assay. Cells with pre-bound POS were incubated at 37 °C for 3 h with serum- and ligand-free DMEM with DMSO solvent (control), or ROCK inhibitor (ROCK inh) as indicated. (**A**,**D**) Cells were fixed without permeabilization and surface-bound POS were labeled with rhodopsin antibody. Fields are representative maximum projections showing an overlay of signals of bound POS (green) and total POS (red). Scale bars, 10 μm. (**B**,**E**) Bars show quantification from images as in **A**,**D** displayed as the fraction of POS signal that did not co-stain with antibody labeling, which indicates internalized POS (red only). Data are presented as mean ± SD from three independent experiments each with five random fields of at least 35 cells counted. ** indicates *p* < 0.01, * indicates *p* < 0.05 according to Student’s *t*-test for pairwise comparison of averages from each experiment (*n* = 3). (**C**,**F**) LysoTracker was added to the final 15 min of phagocytosis incubation followed by live imaging. Representative fields from four independent experiments for each sample type are shown as an overlay of Texas Red POS (red) and LysoTracker (green) channels. Yellow overlay of signals indicates phagosome acidification. Scale bars, 5 μm.

We then used the same overall experimental design but tested POS internalization and POS phagolysosome acidification following a 3 h incubation at a permissive temperature after POS binding. ROCK inhibitor increased POS internalization by wt cells deprived of MerTK ligand ~6-fold on average (Figure 5A,B). Moreover, the labeling of internalized POS with LysoTracker indicated that internalized POS resided in acidified phagolysosomes, implying the successful fusion of lysosomes with engulfed POS (Figure 5C). Strikingly, ROCK inhibitor treatment also increased POS internalization by MerTK-deficient RCS RPE cells, by 2.6-fold on average (Figure 5D,E). Like wt RPE cells, MerTK-deficient RPE cells were able to acidify internalized POS (Figure 5F).

Taken together, acute ROCK inhibition at the time of POS phagocytosis is sufficient to fully rescue the phagocytic defect of RPE cells lacking either MerTK ligand or MerTK receptors.

### 3.5. ROCK Inhibition Promotes Phagocytosis of Endogenous POS by MerTK-Deficient RCS RPE Cells Ex Vivo

**Figure 6:**
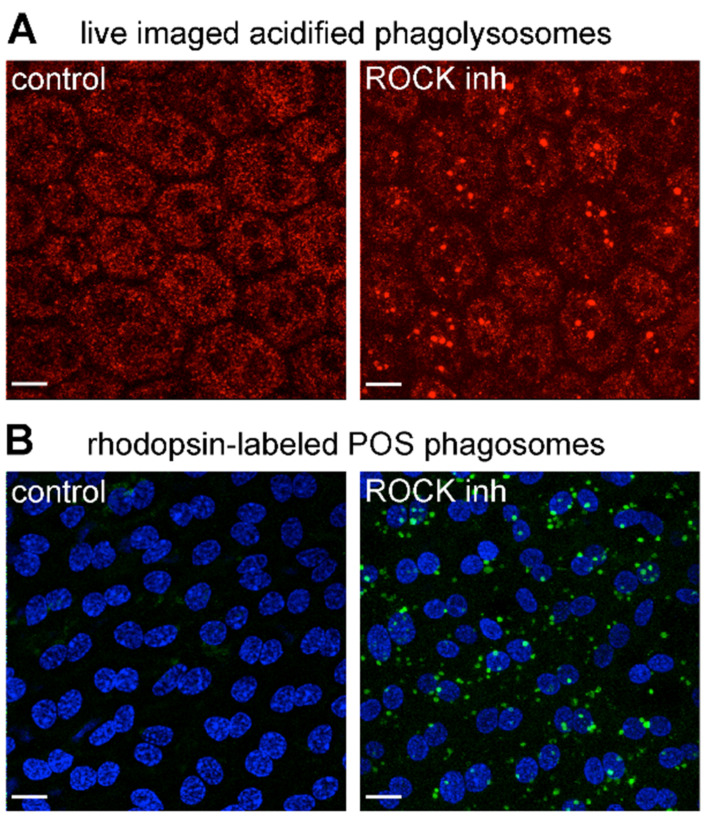
ROCK inhibition is sufficient to promote internalization and acidification of endogenous POS by MerTK receptor-deficient RCS RPE cells ex vivo. Freshly dissected posterior eyecups were incubated for 2 h in DMEM with solvent (control) or DMEM with ROCK inhibitor (ROCK inh) before live LysoTracker labeling of acidified phagosomes (**A**) or washing, fixation and labeling of phagocytosed POS with rhodopsin antibody (**B**). Fields show representative images of four independent experiments with 2 rats for each experiment and eyecups from contralateral eyes used for control and ROCK inhibitor treatments, respectively. Maximal projections are shown of live LysoTracker (**A**, red) and immunofluorescence fluorescence of POS-opsin (**B**, green) with nuclei counterstain (**B**, blue). Scale bars, 10 μm.

Finally, we set out to test if ROCK inhibitor treatment can stimulate POS engulfment by MerTK-defective RPE cells in situ that had not been exposed to the massive changes of the cell culture environment. To this end, we first tested whether the uptake of fluorescent exogenous POS added to freshly dissected posterior eyecups was affected by ROCK inhibitor. However, these trials revealed that the extensive extracellular matrix (likely from the original interphotoreceptor matrix) at the apical surface of the RPE flatmount samples prevented particles from making contact with the RPE cells. Added POS particles remained separated from RPE microvilli by about 2 μm), and, as expected, no engulfment was observed (data not shown). Thus, we instead optimized our retinal dissection protocol to remove the neural retina from the underlying RPE but leaving behind some endogenous outer segments or outer segment debris that were sheared off during the dissection process. We then incubated the resulting posterior eyecups with or without ROCK inhibitor before Lysotracker live labeling and imaging of the RPE. We observed acidified compartments of 1–1.6 μm diameter stained with LysoTracker only in ROCK-inhibitor-treated RCS RPE cells (Figure 6A). These acidified compartments were of the expected size as POS phagolysosomes and identical in appearance to POS phagolysosomes we described previously for WT RPE cells [28]. Furthermore, fixation and immunofluorescence staining after ROCK inhibitor incubation revealed vesicular compartments positive for the POS marker rhodopsin only in RPE tissue that had received ROCK inhibitor, but not in tissue treated with the control medium (Figure 6B). These findings indicate that ROCK inhibition is sufficient to promote the engulfment and acidification of endogenous POS by RPE cells in their native tissue context.

## 4. Discussion

This study identifies the RhoA-ROCK pathway and its effect on F-actin phagocytic cup assembly as the essential and sufficient element of MerTK downstream signaling in RPE phagocytosis, a specialized form of efferocytosis. Our data constitute proof of principle results that the manipulation of cytosolic signaling can bypass MerTK receptors to fully rescue POS engulfment by RPE cells lacking MerTK signaling.

Earlier studies suggest that downstream signaling pathways stimulated by MerTK ligation during clearance phagocytosis are complex. Phosphorylated tyrosines on the activation loop of the MerTK kinase domain may serve as docking sites for cytosolic signaling molecules with an Src-homology 2 (SH2) domain. Phospholipase C, also with an SH2 domain, is another MerTK downstream effector [26]. Moreover, Shelby and colleagues identified numerous cytosolic signaling proteins, many with SH2 domains that may physically or functionally interact with MerTK, including Grb2, PI3KR1, Vav3, c-Src, and Rab-GDI1 [25,38]. These candidates all may influence POS phagocytosis by RPE cells, although it is not yet established to what extent their activities are abnormal in MerTK-deficient RPE cells. Additionally, there is evidence in the literature that silencing MerTK induces RhoA activation and F-actin stress fiber formation, although not in the context of efferocytosis [39]. Despite this plethora of insight into MerTK downstream signaling, there are no published reports, to our knowledge, that manipulating cytosolic signaling elements associated with MerTK signaling is sufficient to rescue MerTK deficiency. Here, we show that the inhibition of ROCK downstream of MerTK and RhoA is both sufficient and required for the engulfment of surface-tethered POS by RPE cells. Any additional signaling protein activated downstream of MerTK during POS phagocytosis will therefore likely either be upstream of or directly affected by ROCK inhibition during phagocytosis and thus either be irrelevant or changed in activity in response to ROCK inhibition, respectively. Alternatively, some of the signaling pathways induced by ligated MerTK may not be essential for POS engulfment. Further studies on the signaling pathways mentioned above will be needed to distinguish these possibilities.

Our data demonstrate that the inhibition of RhoA/ROCK is obligatory for POS engulfment and is promoted by MerTK signaling towards RhoA/ROCK. The highly related TAM receptor Tyro3 can substitute for MerTK, suggesting that it, too, promotes RhoA/ROCK pathway inhibition [24]. In contrast, transgenic overexpression in *mertk*^−/−^ mice of the efferocytosis receptor brain-specific angiogenesis inhibitor 1 (BAI-1) fails to rescue POS phagocytosis despite the ability of BAI-1 to recognize the engulfment signal phosphatidylserine, which is shared by apoptotic cells and POS [11,40]. Based on our data, we speculate that BAI-1 cannot substitute for MerTK function in RPE phagocytosis because it may fail to inhibit RhoA/ROCK upon the engagement of phosphatidylserine-exposing phagocytic particles.

The importance of F-actin phagocytic cups for phagocytosis has long been established [27,41,42]. Not unexpectedly, the global inhibition of all Rho family GTPases by the bacterial toxin B inhibits phagocytic cup formation and engulfment of apoptotic cells by macrophages [43]. An inhibitory role for RhoA-ROCK and requirement for Rac1 activation during apoptotic cell phagocytosis by fibroblast and macrophage cell lines was demonstrated earlier, but these studies did not establish how either of these mechanisms relate to cell surface receptors or phagocytic cup assembly [44,45]. In fibroblast efferocytosis, MerTK has been reported to control Rac1 activity via focal adhesion kinase (FAK) [46]. In contrast, in RPE cells, Rac1 activation, which is essential for F-actin phagocytic cup formation and POS engulfment, depends on αvβ5 integrin but occurs normally in mutant RPE cells lacking MerTK and in RPE cells in which either FAK or MerTK are acutely inhibited [14]. Moreover, a lack of MerTK has no effect on αvβ5 integrin-dependent FAK activation in RPE cells [12]. While prior studies thus imply that the mechanisms of RPE phagocytosis are highly related but not identical to efferocytosis by other cell types, we did not expect to find that activated Rac1 in MerTK-deficient RPE cells is insufficient to induce phagocytic cup formation and POS internalization (Figure 1). While RhoA/ROCK inhibition can increase Rac1 activity [47], it is unlikely to target Rac1 during RPE phagocytosis, as Rac1 activation is normal in MerTK-deficient RPE cells. RhoA/ROCK inhibition may directly increase cofilin activity and/or decrease myosin activity, reducing F-actin stability and cortical actomyosin activity while promoting F-actin branching and stronger membrane curvature [48,49]. Rigidity generated by cortical F-actin limits not only cell membrane branching and curving, but also cell–cell interaction, as observed in the immunological synapse between T-cells and antigen presenting cells [50]. Our data suggest that, in the RPE-POS synapse, reduced cortical tension caused by RhoA-ROCK pathway inactivation may promote membrane extension-based phagocytic cup structures. Full understanding of the molecular effectors of RhoA/ROCK inhibition and Rac1 activation during RPE phagocytosis will require further work that is beyond the scope of the current study.

MerTK mutations cause severe forms of retinitis pigmentosa with childhood onset and complete blindness in early adulthood. Widely applicable therapy remains unavailable to date. Gene therapy aiming to express functional MerTK in the RPE cells using recombinant viruses injected subretinally have had limited success to date [51,52]. Gene correction has been established for RPE cells in culture but has not yet been shown to succeed in vivo [53,54]. Even if gene therapy or correction can be optimized, their in vivo application will require surgery with its high expenses and risks for complications. Ramsden and colleagues reported that compounds promoting translational read-through promote limited full-length MerTK expression in MerTK-mutant ipSC-RPE cells in culture, which leads to a small but significant increase in POS uptake [55]. It remains to be established whether such an approach can be optimized to be effective in vivo, efficient, and safe for long-term application. Our studies show that RPE phagocytosis due to MerTK deficiency can be rescued simply by manipulating MerTK downstream signaling pathways, bypassing the need for receptor restoration. ROCK inhibitors are already approved in common, long-term use for ocular disease (recently reviewed in [56]). In addition to abolishing phagocytosis, MerTK deficiency in the eye also elicits harmful retinal inflammation, which can be alleviated with the application of anti-inflammatory drugs such as eye drops, which slow retinal degeneration in vivo [57]. Altogether, our data well justify future studies toward simple and safe therapy for retinal disease due to MerTK deficiency, combining anti-inflammatory with ROCK inhibitor therapies possibly as eye drop formulations for safe, local, non-invasive therapy.

## Data Availability

Not applicable.

## Supplementary Materials

The following are available online at https://www.mdpi.com/article/10.3390/cells10081927/s1, Figure S1: ROCK inhibitors Y-27632, H 1152, and HA 1100 increase POS internalization by wt RPE cells deprived of MerTK ligand, Figure S2: Cortical F-actin of cells illustrates cell distribution in samples of primary RPE cells as used in this study, Figure S3: Uncut/unprocessed western blot membrane images used to generate Figure 2, Figure S4: Inhibition of ROCK has no effect on POS binding by either wt or MerTK-deficient RCS RPE cells, Figure S5: Uncut/unprocessed western blot membrane image used to generate Supplementary Figure S4.

## References

[1] Young R.W. (1967). The renewal of photoreceptor cell outer segments. J. Cell Biol..

[2] Young R.W., Bok D. (1969). Participation of the retinal pigment epithelium in the rod outer segment renewal process. J. Cell Biol..

[3] Sparrow J.R., Boulton M. (2005). RPE lipofuscin and its role in retinal pathobiology. Exp. Eye Res..

[4] Donato L., Abdalla E., Scimone C., Alibrandi S., Rinaldi C., Nabil K., D’Angelo R., Sidoti A. (2021). Impairments of photoreceptor outer segments renewal and phototransduction due to a peripherin rare haplotype variant: Insights from molecular modeling. Int. J. Mol. Sci..

[5] Donato L., Scimone C., Alibrandi S., Pitruzzella A., Scalia F., D’Angelo R., Sidoti A. (2020). Possible A2E mutagenic effects on RPE mitochondrial DNA from innovative RNA-seq bioinformatics pipeline. Antioxidants.

[6] Yu C., Muñoz L.E., Mallavarapu M., Herrmann M., Finnemann S.C. (2019). Annexin A5 regulates surface αvβ5 integrin for retinal clearance phagocytosis. J. Cell Sci..

[7] Donato L., Scimone C., Alibrandi S., Abdalla E.M., Nabil K.M., D’Angelo R., Sidoti A. (2020). New omics–derived perspectives on retinal dystrophies: Could ion channels-encoding or related genes act as modifier of pathological phenotype?. Int. J. Mol. Sci..

[8] Johansson J.K., Karema-Jokinen V.I., Hakanen S., Jylhä A., Uusitalo H., Vihinen-Ranta M., Skottman H., Ihalainen T.O., Nymark S. (2019). Sodium channels enable fast electrical signaling and regulate phagocytosis in the retinal pigment epithelium. BMC Biol..

[9] Karl M.O., Kroeger W., Wimmers S., Milenkovic V.M., Valtink M., Engelmann K., Strauss O. (2008). Endogenous Gas6 and Ca^2+^-channel activation modulate phagocytosis by retinal pigment epithelium. Cell. Signal..

[10] Müller C., Gómez N.M., Ruth P., Strauß O. (2014). CaV1.3 L-type channels, maxiK Ca^2+^-dependent K+ channels and bestrophin-1 regulate rhythmic photoreceptor outer segment phagocytosis by retinal pigment epithelial cells. Cell. Signal..

[11] Ruggiero L., Connor M.P., Chen J., Langen R., Finnemann S.C. (2012). Diurnal, localized exposure of phosphatidylserine by rod outer segment tips in wild-type but not Itgb5^−/−^ or Mfge8^−/−^ mouse retina. Proc. Natl. Acad. Sci. USA.

[12] Finnemann S.C. (2003). Focal adhesion kinase signaling promotes phagocytosis of integrin-bound photoreceptors. EMBO J..

[13] Finnemann S.C., Bonilha V.L., Marmorstein A.D., Rodriguez-Boulan E. (1997). Phagocytosis of rod outer segments by retinal pig-ment epithelial cells requires αvβ5 integrin for binding but not for internalization. Proc. Natl. Acad. Sci. USA.

[14] Mao Y., Finnemann S.C. (2012). Essential diurnal Rac1 activation during retinal phagocytosis requires αvβ5 integrin but not tyrosine kinases focal adhesion kinase or Mer tyrosine kinase. Mol. Biol. Cell.

[15] Nandrot E.F., Kim Y., Brodie S., Huang X., Sheppard D., Finnemann S.C. (2004). Loss of synchronized retinal phagocytosis and age-related blindness in mice lacking αvβ5 integrin. J. Exp. Med..

[16] Burstyn-Cohen T., Lew E.D., Traves P.G., Burrola P.G., Hash J.C., Lemke G. (2012). Genetic dissection of TAM receptor-ligand interaction in retinal pigment epithelial cell phagocytosis. Neuron.

[17] Audo I., Mohand-Said S., Boulanger-Scemama E., Zanlonghi X., Condroyer C., Démontant V., Boyard F., Antonio A., Méjécase C., El Shamieh S. (2018). MERTK mutation update in inherited retinal diseases. Hum. Mutat..

[18] Gal A., Li Y., Thompson D., Weir J., Orth U., Jacobson S., Apfelstedt-Sylla E., Vollrath D. (2000). Mutations in MERTK, the human orthologue of the RCS rat retinal dystrophy gene, cause retinitis pigmentosa. Nat. Genet..

[19] Parinot C., Nandrot E.F. (2015). A comprehensive review of mutations in the MERTK proto-oncogene. Adv. Exp. Med. Biol..

[20] D’Cruz P.M., Yasumura D., Weir J., Matthes M.T., Abderrahim H., LaVail M.M., Vollrath D. (2000). Mutation of the receptor tyro-sine kinase gene Mertk in the retinal dystrophic RCS rat. Hum. Mol. Genet..

[21] Nandrot E., Dufour E.M., Provost A.C., Pequignot M.O., Bonnel S., Gogat K., Marchant D., Rouillac C., Sepulchre de Conde B., Bihoreau M.T. (2000). Homozygous deletion in the coding sequence of the c-mer gene in RCS rats unravels general mech-anisms of physiological cell adhesion and apoptosis. Neurobiol. Dis..

[22] Bok D., Hall M.O. (1971). The role of the pigment epithelium in the etiology of inherited retinal dystrophy in the rat. J. Cell Biol..

[23] Duncan J.L., Lavail M.M., Yasumura U., Matthes M.T., Yang H., Trautmann N., Chappelow A.V., Feng W., Earp H.S., Matsushima G.K. (2003). An RCS-like retinal dystrophy phenotype in MerKnockout mice. Investig. Opthalmol. Vis. Sci..

[24] Vollrath D., Yasumura D., Benchorin G., Matthes M.T., Feng W., Nguyen N.M., Sedano C.D., Calton M.A., Lavail M.M. (2015). Tyro3 modulates Mertk-associated retinal degeneration. PLoS Genet..

[25] Shelby S.J., Colwill K., Dhe-Paganon S., Pawson T., Thompson D. (2013). MERTK interactions with SH2-domain proteins in the retinal pigment epithelium. PLoS ONE.

[26] Todt J.C., Hu B., Curtis J.L. (2004). The receptor tyrosine kinase MerTK activates phospholipase C gamma2 during recognition of apoptotic thymocytes by murine macrophages. J. Leukoc. Biol..

[27] Mao Y., Finnemann S.C. (2015). Regulation of phagocytosis by Rho GTPases. Small GTPases.

[28] Mao Y., Finnemann S.C. (2015). Live imaging of LysoTracker-labelled phagolysosomes tracks diurnal phagocytosis of photoreceptor outer segment fragments in Rat RPE tissue ex vivo. Adv. Exp. Med. Biol..

[29] Adamus G., Zam Z.S., Arendt A., Palczewski K., McDowell J.H., Hargrave P.A. (1991). Anti-rhodopsin monoclonal antibodies of defined specificity: Characterization and application. Vis. Res..

[30] Mazzoni F., Dun Y., Vargas J.A., Nandrot E.F., Finnemann S.C. (2021). Lack of the antioxidant enzyme methionine sulfoxide reductase A in mice impairs RPE phagocytosis and causes photoreceptor cone dysfunction. Redox Biol..

[31] Parinot C., Rieu Q., Chatagnon J., Finnemann S.C., Nandrot E.F. (2014). Large-Scale Purification of Porcine or Bovine Photoreceptor Outer Segments for Phagocytosis Assays on Retinal Pigment Epithelial Cells. J. Vis. Exp..

[32] Mazzoni F., Mao Y., Finnemann S.C. (2018). Advanced analysis of photoreceptor outer segment phagocytosis by RPE cells in culture. Methods Mol. Biol..

[33] Finnemann S.C., Rodriguez-Boulan E. (1999). Macrophage and retinal pigment epithelium phagocytosis: Apoptotic cells and pho-toreceptors compete for αvβ3 and αvβ5 integrins, and protein kinase C regulates αvβ5 binding and cytoskeletal linkage. J. Exp. Med..

[34] Schmidt G., Sehr P., Wilm M., Selzer J., Mann M., Aktories K. (1997). Gln 63 of Rho is deamidated by Escherichia coli cytotoxic necrotizing factor-1. Nat. Cell Biol..

[35] Uehata M., Ishizaki T., Satoh H., Ono T., Kawahara T., Morishita T., Tamakawa H., Yamagami K., Inui J., Maekawa M. (1997). Calcium sensitization of smooth muscle mediated by a Rho-associated protein kinase in hypertension. Nat. Cell Biol..

[36] Moolenaar W.H., van Meeteren L., Giepmans B. (2004). The ins and outs of lysophosphatidic acid signaling. Bioessays.

[37] Schoenwaelder S.M., Burridge K. (1999). Evidence for a calpeptin-sensitive protein-tyrosine phosphatase upstream of the small GTPase rho. A novel role for the calpain inhibitor calpeptin in the inhibition of protein-tyrosine phosphatases. J. Biol. Chem..

[38] Shelby S.J., Feathers K.L., Ganios A.M., Jia L., Miller J., Thompson D. (2015). MERTK signaling in the retinal pigment epithelium regulates the tyrosine phosphorylation of GDP dissociation inhibitor alpha from the GDI/CHM family of RAB GTPase effectors. Exp. Eye Res..

[39] Rogers A.E.J., Le J.P., Sather S., Pernu B.M., Graham D.K., Pierce A.M., Keating A.K. (2011). Mer receptor tyrosine kinase inhibition impedes glioblastoma multiforme migration and alters cellular morphology. Oncogene.

[40] Penberthy K.K., Rival C., Shankman L.S., Raymond M.H., Zhang J., Perry J.S.A., Lee C.S., Han C.Z., Onengut-Gumuscu S., Palczewski K. (2017). Context-dependent compensation among phosphatidylserine-recognition receptors. Sci. Rep..

[41] Allen L.-A., Aderem A. (1996). Mechanisms of phagocytosis. Curr. Opin. Immunol..

[42] Chimini G., Chavrier P. (2000). Function of Rho family proteins in actin dynamics during phagocytosis and engulfment. Nat. Cell Biol..

[43] Leverrier Y., Ridley A. (2001). Requirement for Rho GTPases and PI 3-kinases during apoptotic cell phagocytosis by macrophages. Curr. Biol..

[44] Nakaya M., Tanaka M., Okabe Y., Hanayama R., Nagata S. (2006). Opposite effects of Rho family GTPases on engulfment of apoptotic cells by macrophages. J. Biol. Chem..

[45] Tosello-Trampont A.-C., Nakada-Tsukui K., Ravichandran K.S. (2003). Engulfment of apoptotic cells is negatively regulated by Rho-mediated signaling. J. Biol. Chem..

[46] Wu Y., Singh S., Georgescu M.-M., Birge R.B. (2005). A role for Mer tyrosine kinase in αvβ5 integrin-mediated phagocytosis of apoptotic cells. J. Cell Sci..

[47] Tsuji T., Ishizaki T., Okamoto M., Higashida C., Kimura K., Furuyashiki T., Arakawa Y., Birge R.B., Nakamoto T., Hirai H. (2002). ROCK and mDia1 antagonize in Rho-dependent Rac activation in Swiss 3T3 fibroblasts. J. Cell Biol..

[48] Maekawa M., Ishizaki T., Boku S., Watanabe N., Fujita A., Iwamatsu A., Obinata T., Ohashi K., Mizuno K., Narumiya S. (1999). Signaling from Rho to the actin cytoskeleton through protein kinases ROCK and LIM-kinase. Science.

[49] Narumiya S., Thumkeo D. (2018). Rho signaling research: History, current status and future directions. FEBS Lett..

[50] Faure S., Salazar-Fontana L.I., Semichon M., Tybulewicz V., Bismuth G., Trautmann A., Germain R.N., Delon J. (2004). ERM proteins regulate cytoskeleton relaxation promoting T cell–APC conjugation. Nat. Immunol..

[51] Ghazi N., Abboud E.B., Nowilaty S.R., Alkuraya H., Alhommadi A., Cai H., Hou R., Deng W.-T., Boye S.L., Almaghamsi A. (2016). Treatment of retinitis pigmentosa due to MERTK mutations by ocular subretinal injection of adeno-associated virus gene vector: Results of a phase I trial. Qual. Life Res..

[52] Garafalo A.V., Cideciyan A.V., Héon E., Sheplock R., Pearson A., Yu C.W., Sumaroka A., Aguirre G.D., Jacobson S.G. (2020). Progress in treating inherited retinal diseases: Early subretinal gene therapy clinical trials and candidates for future initiatives. Prog. Retin. Eye Res..

[53] Castro A.A., Long K., Bassett A., Machuca C., León M., Ávila-Fernandez A., Corton M., Vidal-Puig T., Ayuso C., Lukovic D. (2019). Generation of gene-corrected human induced pluripotent stem cell lines derived from retinitis pigmentosa patient with Ser331Cysfs*5 mutation in MERTK. Stem Cell Res..

[54] Artero-Castro A., Long K., Bassett A., Ávila-Fernandez A., Cortón M., Vidal-Puig A., Jendelova P., Rodriguez-Jimenez F., Clemente E., Ayuso C. (2021). Gene correction recovers phagocytosis in retinal pigment epithelium derived from retinitis pigmentosa-human-induced pluripotent stem cells. Int. J. Mol. Sci..

[55] Ramsden C.M., Nommiste B., Lane A.R., Carr A.-J.F., Powner M.B., Smart M.J.K., Chen L.L., Muthiah M.N., Webster A.R., Moore A.T. (2017). Rescue of the MERTK phagocytic defect in a human iPSC disease model using translational read-through inducing drugs. Sci. Rep..

[56] Al-Humimat G., Marashdeh I., Daradkeh D., Kooner K. (2021). Investigational Rho kinase inhibitors for the treatment of glaucoma. J. Exp. Pharmacol..

[57] Lew D.S., Mazzoni F., Finnemann S.C. (2020). microglia inhibition delays retinal degeneration due to MerTK phagocytosis receptor deficiency. Front. Immunol..

