# Supplementary material for: Acute RhoA/Rho Kinase Inhibition Is Sufficient to Restore Phagocytic Capacity to Retinal Pigment Epithelium Lacking the Engulfment Receptor MerTK
Source: Cells. 2021 Jul 29;10(8):1927. doi: 10.3390/cells10081927 (PMC8394172; doi:10.3390/cells10081927)
Supplement: Supplementary file 1 [file cells-10-01927-s001.zip › cells-1278482-supplementary.pdf]

Mao and Finnemann

Acute RhoA/Rho Kinase inhibition is sufficient to restore phagocytic capacity to retinal pigment epithelium lacking the engulfment receptor MerTK

Supplementary Figures S1 – S5

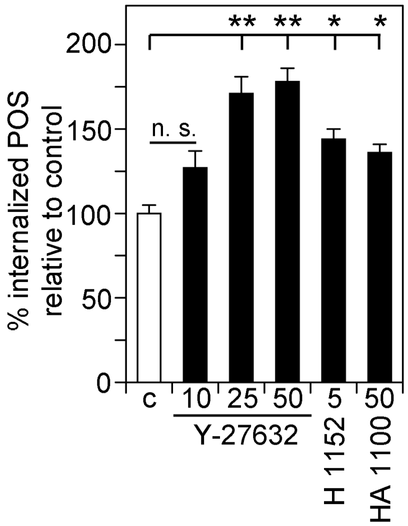

Supplementary Figure S1.

ROCK inhibitors Y-27632, H 1152, and HA 1100 increase POS internalization by wt RPE cells deprived of MerTK ligand.

Wt rat RPE cells were treated with Texas Red labeled POS in DMEM supplemented with MFG-E8 and with DMSO solvent only as control (c) or different ROCK inhibitors at concentrations as indicated for 3 hours followed by surface labeling of bound POS with rhodopsin antibody and AlexaFluor488 conjugated secondary antibody. Final DMSO concentration was 0.5% for all samples. Bars show amount of internalized POS material (Texas Red positive, Alexafluor488 negative) relative to internalized POS by control cells treated with solvent, which was set as 100%; mean ± SD ; n = 3 independent experiments. ** indicates $p < 0.01$, * indicates $p < 0.05$ according to the Kruskal-Wallis test and Dunn’s post-hoc test; n. s., not significant, $p > 0.05$.

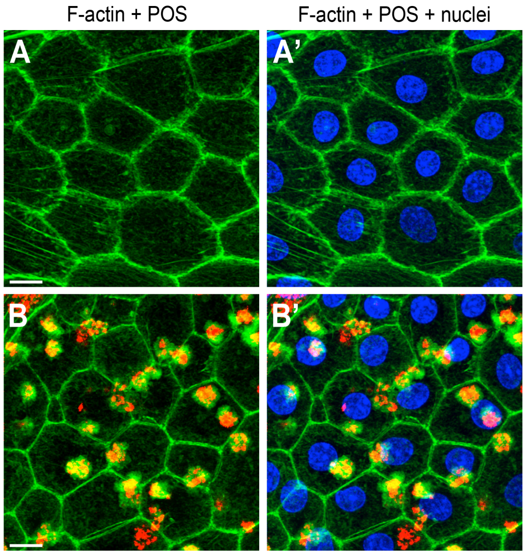

Supplementary Figure S2.

Cortical F-actin of cells illustrates cell distribution in samples of primary RPE cells as used in this study.

Primary wt rat RPE cells were challenged with DMEM (**A**, **A'**) or Texas Red-labeled POS (**B**, **B'**) in a synchronized phagocytosis assay. Following DMEM or POS challenge, washed cells were further incubated at 37°C for 10 min with DMEM supplemented with Protein S before fixation and F-actin labeling. Fields show projections of maximal overlays of F-actin (green) and POS (red) (**A**, **B**), or of F-actin (green), POS (red), and nuclei (blue) (**A'**, **B'**). Scale bars, 10 µm.

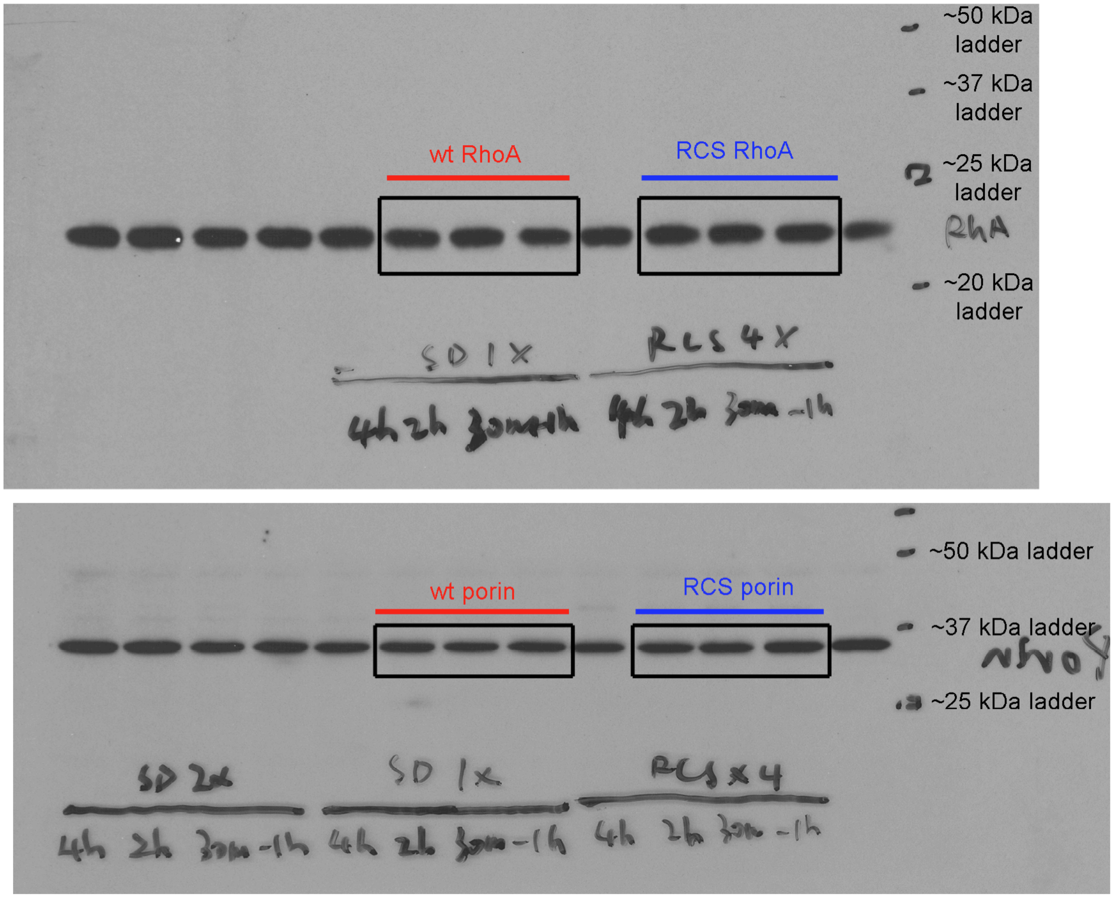

Supplementary Figure S3.

Uncut/unprocessed western blot membrane images used to generate Figure 2.

Lanes used to generate panels for Figure 2 are indicated by black boxes. All other lanes show samples from experimental repeats that were not used to generate the images shown in Figure 2. Note that protein levels at the 4-hour time point were not included in Figure 2, as we did not perform RhoA activity assays at this time point.

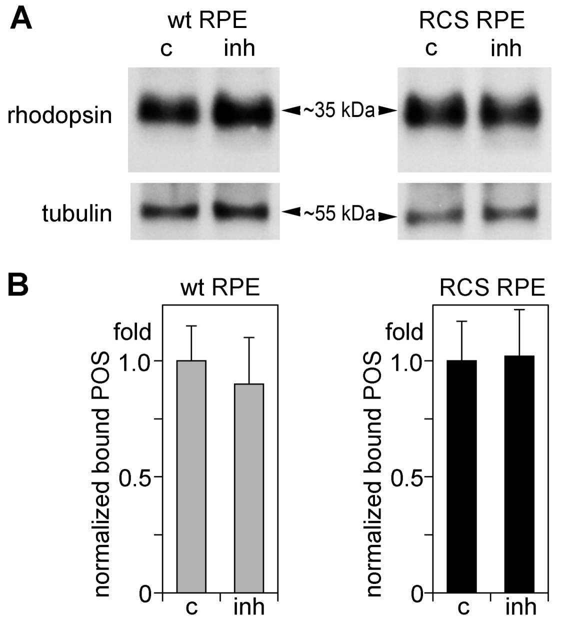

Supplementary Figure S4.

Inhibition of ROCK has no effect on POS binding by either wt or MerTK-deficient RCS RPE cells.

Wt and RCS primary rat RPE cells were treated with ROCK inhibitor (inh) or control solvent (c) and POS with MFG-E8 at 20°C for 1 h to allow POS surface binding. Total POS-rhodopsin level in cell lysates was detected by rhodopsin immunoblotting. α-tubulin antibody probing in the same development served to normalize RPE cell load per sample. (**A**) Immunoblots from a representative experiment are shown with approximate molecular sizes of proteins indicated. The uncut blot membrane image is shown in Supplementary Figure 4. (**B**) Bars report ratio of POS-rhodopsin to tubulin with ratio in control solvent treated cells set as 1 for each cell type; mean ± SD; n = 4 independent experiments. Differences were not significant according to Mann-Whitney test, $p > 0.05$. Gray bars: wt RPE; black bars: RCS RPE.

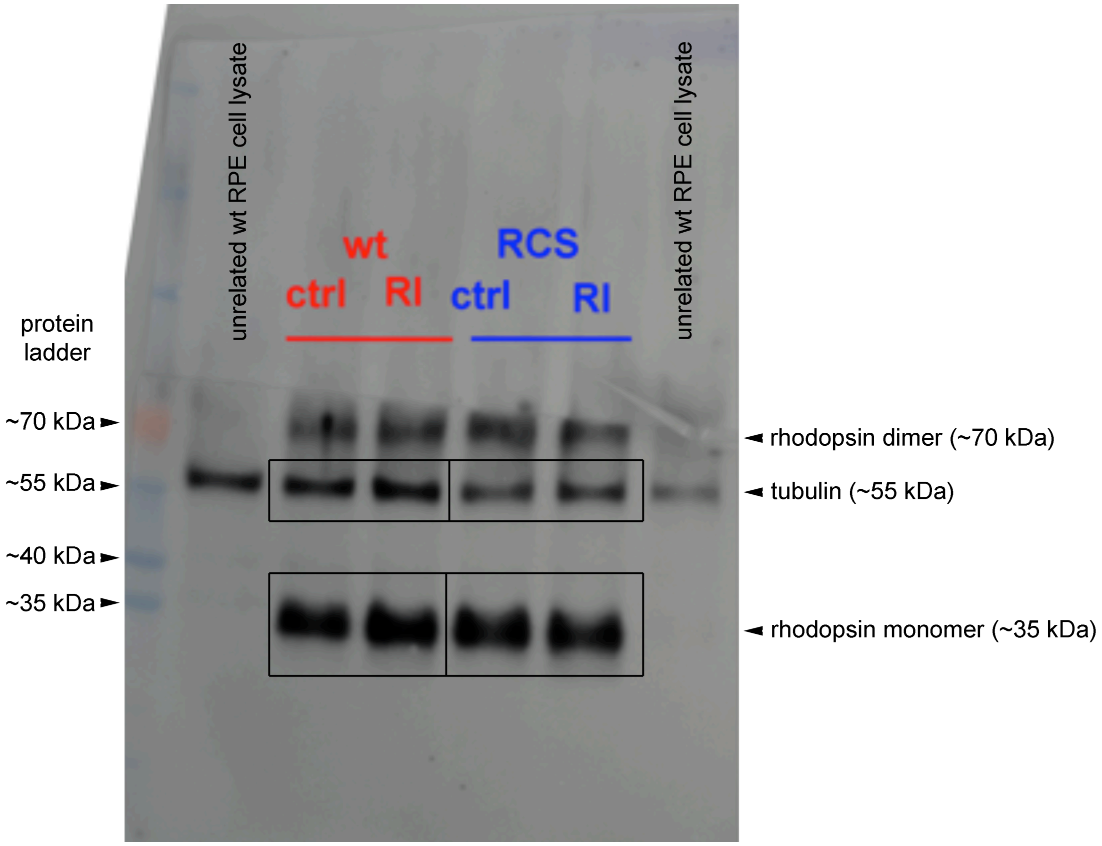

Supplementary Figure S5.

Uncut/unprocessed western blot membrane image used to generate Supplementary Figure 4.

Lanes used to generate panels for Supplementary Figure 3 are indicated by black boxes.
